# Factors influencing stigma in bipolar disorder type I

**DOI:** 10.1192/j.eurpsy.2024.887

**Published:** 2024-08-27

**Authors:** A. Adouni, Y. Zgueb, I. Bouguerra, F. Ben Othman, R. Jomli

**Affiliations:** psychiatry department A, Razi hospital, Manouba, Tunisia

## Abstract

**Introduction:**

Given the recurrence of mood episodes, with their negative repercussions such as high suicidal risk, significant cognitive decline and the persistence of residual signs with a negative impact on the patient’s family, social and professional functioning, Bipolar Disorder is a mental disorder with a significant social stigma.

**Objectives:**

Identify the socio-demographic and clinical factors that may influence the experience of stigma in bipolar disorder type I

**Methods:**

We conducted a cross-sectional, comparative study over a six-month period at the aftercare unit of Razi Hospital’s psychiatric ward “A”, including patients treated for TB I according to DSM 5 criteria and stable on treatment.

The study was conducted in two stages: first, sociodemographic and clinical characteristics were collected using a pre-established form. The DISCUS scale, validated in Arabic, was then administered.

**Results:**

We included 100 patients (60 men and 40 women) with a mean age of 43.55 years.

The median DISCUS stigma score was 6 (0-19).

The mean value of the DISCUS scale was high for patients of urban origin (p=0.042), with a low socioeconomic level (p=0.001), and poor family dynamics (p<0.001).

The presence of a comorbid personality disorder was significantly associated with stigma (p=0.006). The DISCUS scale was positively associated with the number of years of follow-up, the number of hospitalizations, the number of manic episodes, the number of depressive episodes and the number of episodes with psychotic or melancholic features.

**Conclusions:**

This stigma can have a negative impact on patients’ quality of life in a whole range of ways, including limiting their opportunities for education, employment and housing.

Intensive therapeutic interventions should be considered for vulnerable patients to limit the consequences.

**Disclosure of Interest:**

None Declared

